# RBM24 suppresses cancer progression by upregulating miR-25 to target MALAT1 in nasopharyngeal carcinoma

**DOI:** 10.1038/cddis.2016.252

**Published:** 2016-09-01

**Authors:** Wen-Feng Hua, Qian Zhong, Tian-Liang Xia, Qi Chen, Mei-Yin Zhang, Ai-Jun Zhou, Zi-Wei Tu, Chen Qu, Man-Zhi Li, Yun-Fei Xia, Hui-Yun Wang, Dan Xie, Francois-Xavier Claret, Er-Wei Song, Mu-Sheng Zeng

**Affiliations:** 1 State Key Laboratory of Oncology in South China, Sun Yat-sen University Cancer Center, Collaborative Innovation Center of Cancer Medicine, Guangzhou, China; 2 Department of Systems Biology, University of Texas-MD Anderson Cancer Center, Houston, TX, USA; 3 Breast Cancer Center of Sun Yat-sen Memorial Hospital, Sun Yat-sen University, Guangzhou, China

## Abstract

Abnormal interaction between non-coding RNAs has been demonstrated to be a common molecular event in various human cancers, but its significance and underlying mechanisms have not been well documented. RNA-binding proteins (RBPs) are key regulators of RNA transcription and post-transcriptional processing. In this study, we found that RNA-binding protein 24 (RBM24) was frequently downregulated in nasopharyngeal carcinoma (NPC). The restoration of RBM24 expression suppressed NPC cellular proliferation, migration and invasion and impeded metastatic colonization in mouse models. Microarray analyses revealed that miR-25 expression was upregulated by RBM24 expression in NPC cells. Similarly, ectopic miR-25 expression suppressed NPC cellular growth and motility by targeting the pro-oncogenic lncRNA MALAT1, and the knockdown of MALAT1 expression exhibited similar effects as RBM24 restoration in NPC cells. Overall, these findings suggest a novel role of RBM24 as a tumor suppressor. Mechanistically, RBM24 acts at least in part through upregulating the expression of miR-25, which in turn targets MALAT1 for degradation.

Nasopharyngeal carcinoma (NPC) is a highly malignant cancer that often invades adjacent regions and metastasizes to regional lymph nodes and distant organs. Although early-stage NPC is highly radiocurable, the treatment results of locoregionally advanced NPC have been disappointing.^1, 2^ Therefore, elucidation of the molecular mechanisms underlying the tumorigenicity, invasion and metastasis of NPC is very important for the treatment of this disease.

Recently, comprehensive microarray analysis has revealed a microRNA (miRNA) signature that is significantly associated with the prognosis and progression of NPC.^3, 4, 5^ Among the numerous differentially expressed miRNAs in NPC, three miRNAs, including miR-29c, miR-9 and miR-26a, have been shown to be significantly downregulated and have been extensively studied in association with this disease.^5, 6, 7, 8, 9, 10, 11, 12^ Accumulating evidence indicates that long noncoding RNAs (lncRNAs) are frequently deregulated in the malignant transformation and progression of various types of cancer, including NPC.^13, 14, 15, 16, 17, 18^ Nie *et al.* have shown that HOX antisense intergenic RNA (HOTAIR) is significantly upregulated in fresh primary NPC tissues and paraffin-embedded samples compared with non-cancer tissue samples. Moreover, patients with a high *HOTAIR* level have been shown to have a poor clinical outcome, including tumor recurrence and distant metastasis.^19^ In addition, metastasis associated with lung adenocarcinoma transcript 1 (MALAT1) has been demonstrated to be extremely abundant in many human malignancies, including cancers of the prostate, bladder, breast and nasopharynx.^20, 21, 22, 23^ Xie *et al.* have reported that MALAT1 is significantly upregulated in poorly differentiated NPC cell lines, including 5-8 F, CNE-2, C666-1 and HONE-1, while it is downregulated in highly differentiated CNE-1 cells and immortalized NP69 epithelial cells. Functional studies have shown that MALAT1 promotes the proliferation, migration and invasion of CNE-1 cells *in vitro.*^23^ Although the particular molecular mechanism involving MALAT1 is currently under debate, functional studies have confirmed that its deregulation influences the proliferation, invasion and/or metastasis of multiple types of cancer cells.^24^

RBM24, an RNA-binding protein (RBP) with a single conserved RNA recognition motif (RRM) domain, has been found to be preferentially expressed in cardiac and skeletal muscle tissues^25, 26^ and to play important roles in myogenic differentiation and heart development.^26, 27, 28, 29^ Furthermore, recent studies have demonstrated that it may regulate the stability of p21 and p63 mRNA transcripts in different human cancer cell lines.^30, 31^ However, the exact functions of RBM24 in tumorigenesis and cancer progression are largely unknown.

In the present study, we found that RBM24 was downregulated in NPC. The restoration of RBM24 expression in NPC cells suppressed cellular proliferation, motility and invasion. Next, we performed xenograft experiments to show that RBM24 overexpression caused the inhibition of NPC tumorigenesis and conveyed a survival advantage through its propensity to suppress tumor metastasis. Furthermore, we performed miRNA array analysis and found that miR-25 was the most highly upregulated miRNA in response to RBM24 overexpression and that this miRNA silenced MALAT1 in an Argonaute2 (Ago2)-dependent manner. This post-transcriptional regulation may lead to inhibition of the growth, invasion and metastasis of NPC cells. Taken together, our results demonstrate that RBM24 functions as a novel tumor suppressor in NPC by repressing tumorigenicity and invasion. It possibly exerts its tumor suppressor function via increasing the expression of miR-25, which directly targets MALAT1 for degradation.

## Results

### RBM24 is frequently downregulated in NPC

**Figure 1:**
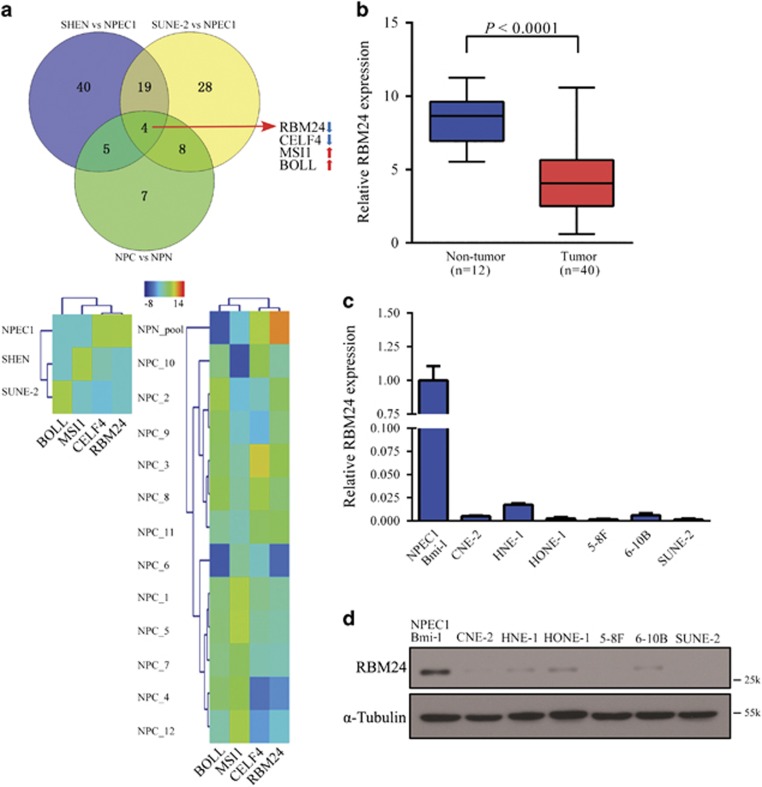
The downregulation of RBM24 in NPC. (**a**) Upper: Venn diagram showing the overlap of RBP genes with different expression levels in NPC cells and tumor samples. Lower: Hierarchical clustering of gene RPKMs of four RBP-encoding genes across NPEC1, NPC cells and patient samples. (**b**) qRT-PCR showing the relative RBM24 expression levels in 40 NPC samples *versus* 12 non-cancerous nasopharyngeal tissue samples. (**c** and **d**) The relative RBM24 mRNA and protein levels in NPC cells, as determined by qRT-PCR and immunoblotting, respectively. The error bars represent the mean±S.E.M. from three independent experiments

To identify the differentially expressed RBP-encoding genes in NPC, we comparatively analyzed mRNA expression profiles of NPC cells and tumor samples from our previous RNA-seq study.^32^ Four common RBP-encoding genes were found to be significantly differentially expressed in both the NPC cells and tumor samples compared with primary NPEC1 cells and non-tumor tissues, respectively (Figure 1a). One of the downregulated genes, RBM24, was further characterized. We performed qRT-PCR analysis using an additional 40 primary NPC samples, 12 non-cancerous nasopharyngeal tissues and 6 NPC cell lines. As shown in Figures 1b and c, RBM24 was significantly downregulated in the tumor tissues and NPC cells compared with the non-tumor tissues and immortalized NPEC1 Bmi-1 cells, respectively. To further verify that the downregulation of RBM24 is a common event in NPC, we retrieved the mRNA expression profiles from two data sets (GSE12452 and GSE53819). The results also showed that RBM24 was significantly downregulated in the tumor tissues compared with non-tumor tissues (Supplementary Figure S1). In addition, western blot revealed that RBM24 protein expression was consistently lower in all six NPC cell lines compared with immortalized NPEC1 Bmi-1 cells (Figure 1d).

### Restoration of RBM24 expression in NPC cells suppressed cellular proliferation, migration and invasion

**Figure 2:**
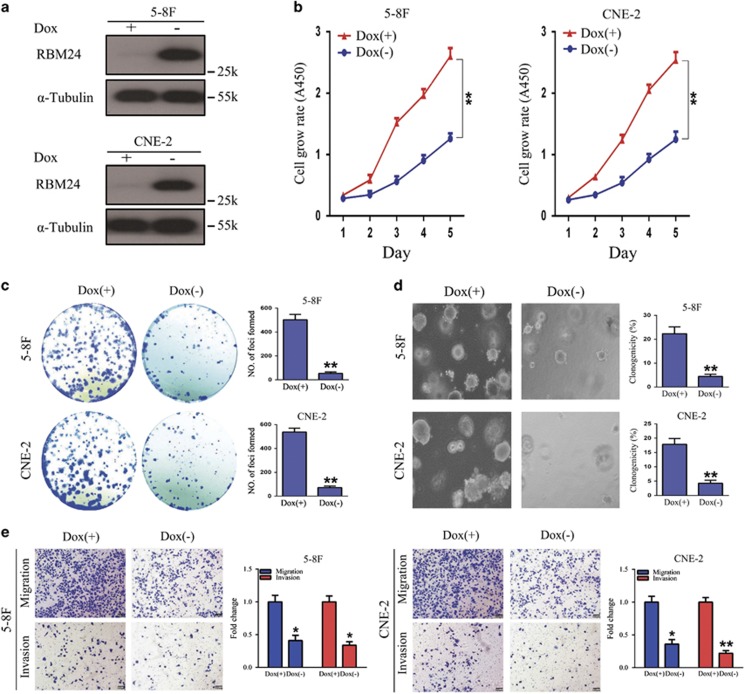
RBM24 suppresses NPC proliferation, migration and invasion *in vitro*. (**a**) Western blotting analysis verifying the overexpression of *RBM24* in 5-8 F and CNE-2 Tet-Off-inducible RBM24-stable cells treated with or without doxycycline. (**b**) The cell growth rates were determined by CCK8 assay (**P<0.01, Student's *t-*test). (**c**) Induction of foci formation and (**d**) colony formation in soft agar of 5-8 F and CNE-2 Tet-Off-inducible RBM24-stable cells treated with or without doxycycline. The numbers of foci and colonies were calculated and are depicted in the bar chart. The values indicate the mean standard deviation of three independent experiments (magnification, × 200; **P<0.01, Student's *t*-test). (**e**) Transwell assay showing the migration and invasion of 5-8 F and CNE-2 Tet-Off-inducible RBM24-stable cells treated with or without doxycycline. Migrating and invaded cells were fixed and stained with crystal violet (magnification, × 100). The number of migrating and invaded cells were calculated and are depicted in the bar chart. All data are shown as the mean±S.E.M. of three independent experiments (*P<0.05 and **P<0.01, Student's *t-*test)

**Figure 3:**
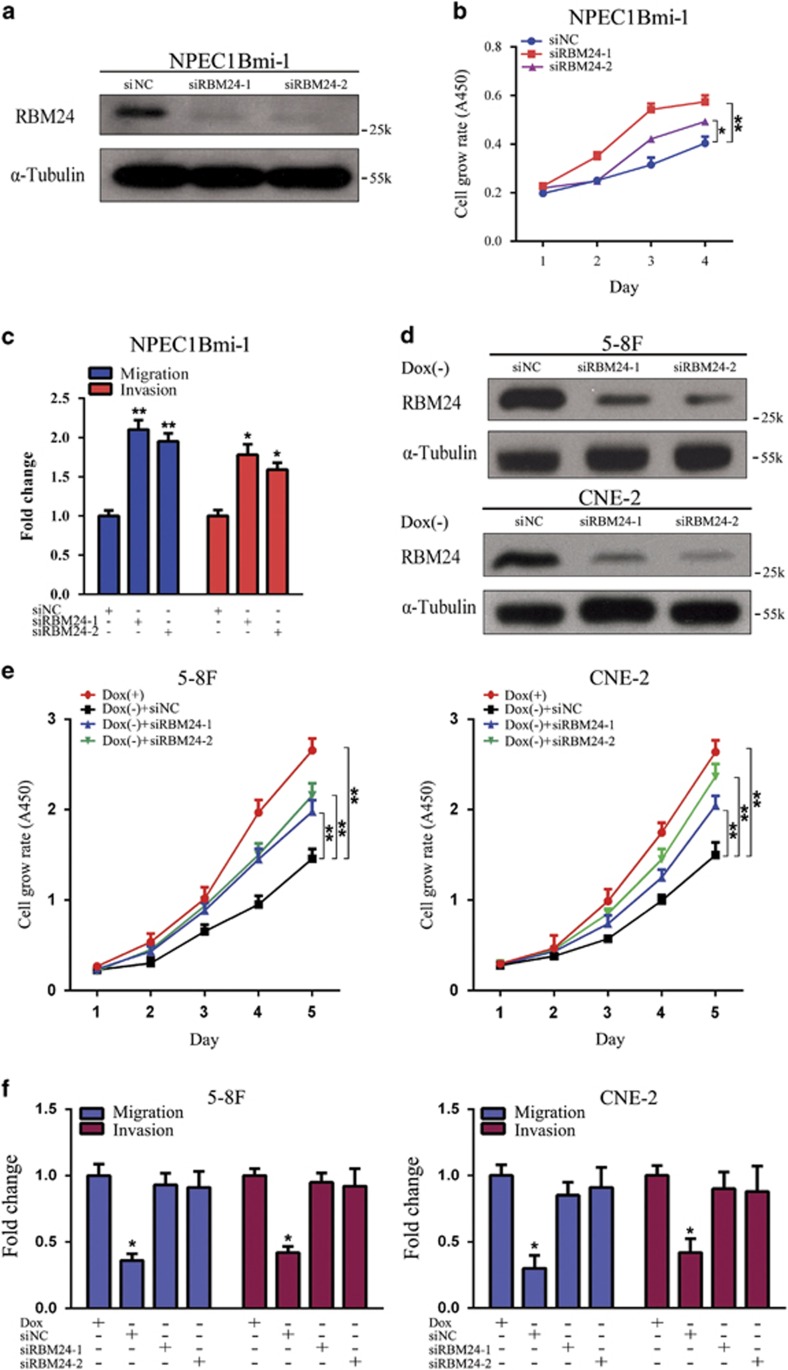
*RBM24* knockdown attenuates the inhibitory effect of RBM24 expression *in vitro*. (**a**) Western blot analysis of RBM24 expression in NPEC1Bmi-1 cells that were transiently transfected with siRNAs (50 nM final concentration) against RBM24 or scramble siRNAs as controls. (**b**) The cell growth rates were determined by CCK8 assay (*P<0.05 and **P<0.01, Student's *t*-test). (**c**) Quantification of the indicated migrating and invaded cells by transwell assay for NPEC1Bmi-1 cells treated as in (**a**) (*P<0.05 and **P<0.01, Student's *t*-test). (**d**) Western blot analysis of RBM24 expression in 5-8 F and CNE-2 Tet-Off-inducible RBM24-stable cells that were transiently transfected with siRBM24 or siNC after the removal of doxycycline for 24 h. (**e**) CCK8 assay of proliferation of 5-8 F and CNE-2 Tet-Off-inducible RBM24-stable cells treated as in (**d**) (**P<0.01, Student's *t-*test). (**f**) Quantification of the indicated migrating and invaded cells by transwell assay for 5-8 F and CNE-2 Tet-Off-inducible RBM24-stable cells treated as in (**d**). All data are shown as the mean±S.E.M. of three independent experiments (*P<0.05 and **P<0.01, Student's *t*-test)

The downregulation of RBM24 in NPC suggests that it might function as a novel tumor suppressor. To assess whether RBM24 possesses a tumor suppressive function, 5-8 F and CNE-2 cells, which expressed the lowest RBM24 levels among all of the tested NPC cell lines, were selected for generation of Tet-Off-inducible RBM24 expression cells, and the induction of RBM24 expression was confirmed by western blotting (Figure 2a). *In vitro* functional assays were performed to investigate the tumor suppressive role of RBM24. The CCK8 assay results indicated that RBM24 substantially reduced the rate of cell proliferation (Figure 2b). Foci formation revealed that RBM24 significantly reduced the frequency of colony formation on solid plates (Figure 2c). A similar result was obtained from soft agar assay, in which the induction of RBM24 expression resulted in a reduction in the colony formation efficiency in 5-8 F and CNE-2 cells from an average of 22.3 to 4.4% and 17.8 to 4.2%, respectively (Figure 2d). These results indicated that it inhibited tumor growth in both anchorage-dependent and -independent manners. We also studied the effects of RBM24 on the migratory and invasive capacities of NPC cells by transwell assays, which showed that they were efficiently suppressed (Figure 2e). To further confirm the tumor suppressive function of RBM24, endogenous RBM24 expression was silenced in an immortalized NPEC1 Bmi-1 cell line using two siRNAs targeting RBM24 (Figure 3a). As expected, the cell proliferation, migration and invasion abilities of NPEC1 Bmi-1 cells were significantly increased following the knock down of RBM24 (Figures 3b and c). Moreover, the silencing of RBM24 in Tet-Off-inducible RBM24-stable cells partly relieved the proliferative inhibitory effect of RBM24 expression and restored the migration and invasion abilities of the cells (Figures 3d–f). Taken together, these findings demonstrate that RBM24 suppresses the proliferation, migration and invasion of NPC cells *in vitro*.

### RBM24 upregulates miR-25 expression in NPC cells

**Figure 4:**
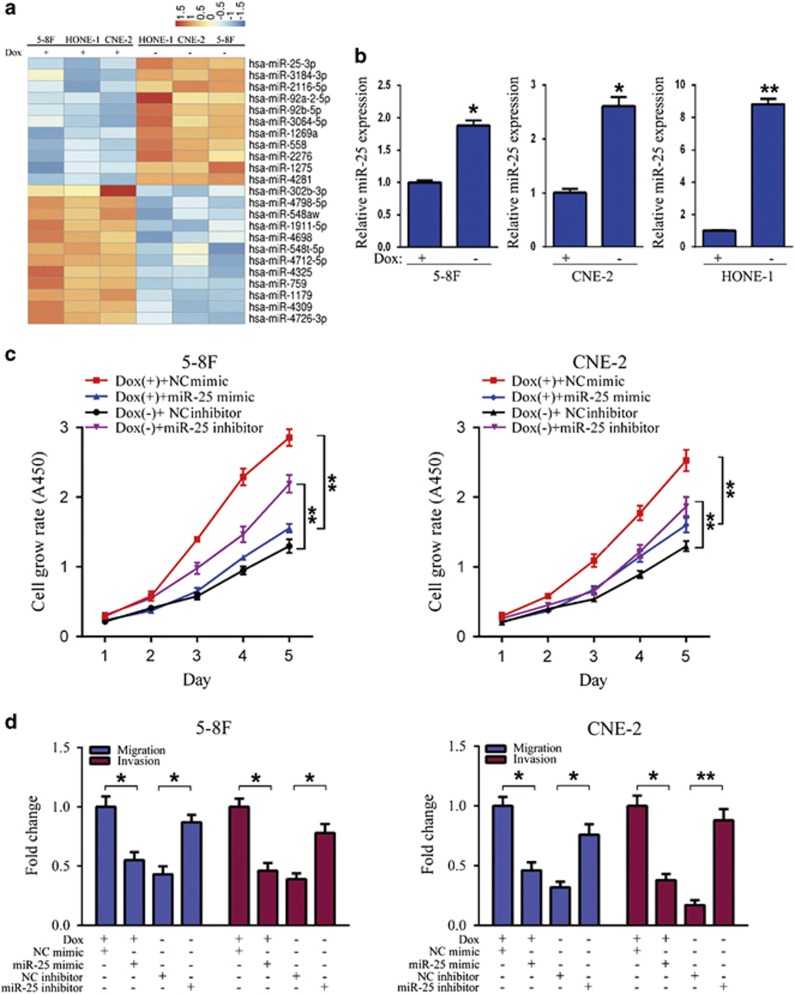
RBM24 upregulates miR-25 expression. (**a**) Microarray profiling results showing the miRNAs that were regulated by RBM24. (**b**) QRT-PCR analysis of miR-25 expression in 5-8 F, CNE-2 and HONE-1 Tet-Off-inducible RBM24-stable cells treated with or without doxycycline (*P<0.05 and **P<0.01, Student's *t-*test). (**c**) CCK8 assay of proliferation of 5-8 F and CNE-2 Tet-Off-inducible RBM24-stable cells that were transfected with miR-25 mimic (50 nM), NC mimic, miR-25 inhibitor (100 nM) or NC inhibitor after treatment with doxycycline or removal of doxycycline for 24 h (**P<0.01, Student's *t-*test). (**d**) Quantification of the indicated migrating and invaded cells by transwell analysis for 5-8 F and CNE-2 Tet-Off-inducible RBM24-stable cells treated as in (**c**). All data are shown as the mean±S.E.M. of three independent experiments (*P<0.05 and **P<0.01, Student's *t*-test)

In search of the underlying mechanisms by which RBM24 inhibits NPC cell proliferation and mobility *in vitro*, we reasoned that miRNAs are critical regulators of these processes because RBPs play important roles in producing miRNAs by promoting the efficient processing of miRNA precursors.^33^ We first compared the miRNA expression profiles of RBM24-induced cells and control cells using a human miRNA microarray. As expected, microarray analysis revealed 11 commonly upregulated and 12 commonly downregulated miRNAs in the RBM24-induced cells (Figure 4a and Supplementary Table S1). These 23 miRNAs were sorted in descending order according to their fold changes, and miR-25 was ranked the highest out of all of the common upregulated genes. Further, qRT-PCR analysis of three Tet-Off-inducible RBM24-stable cells showed that the induction of RBM24 expression significantly increased the miR-25 level (Figure 4b). In addition, qRT-PCR analysis also identified a few others commonly upregulated miRNAs expression was significantly increased with induction of RBM24 expression in the Tet-Off-inducible RBM24-stable cells (Supplementary Figure S2).

### MiR-25 suppresses the proliferation, migration and invasion of NPC cells

The observation that miR-25 expression was upregulated after the induction of RBM24 expression prompted us to further investigate the biological function of miR-25. Tet-Off-inducible RBM24-stable cells were transfected with miR-25 mimics or inhibitor to overexpress or suppress miR-25 expression, respectively. As shown in Figure 4c, CCK8 assay revealed that the ectopic expression of miR-25 slowed the propagation of NPC cells. Conversely, suppression of endogenous miR-25 expression resulted in significant attenuation of the proliferative inhibitory effect of RBM24 expression. Next, we investigated whether miR-25 overexpression has suppressive effects on migration and invasion. Transwell assays revealed that miR-25 overexpression suppressed the migration and invasion abilities of the 5-8 F and CNE-2 Tet-Off-inducible RBM24-stable cells (Figure 4d). In contrast, the miR-25 inhibitor significantly attenuated the inhibitory effects of RBM24 expression on migration and invasion (Figure 4d). Taken together, these data indicate that miR-25 can be considered a downstream effector molecule for the inhibitory effects of RBM24 expression in NPC cells.

### MiR-25 directly targets the lncRNA MALAT1

**Figure 5:**
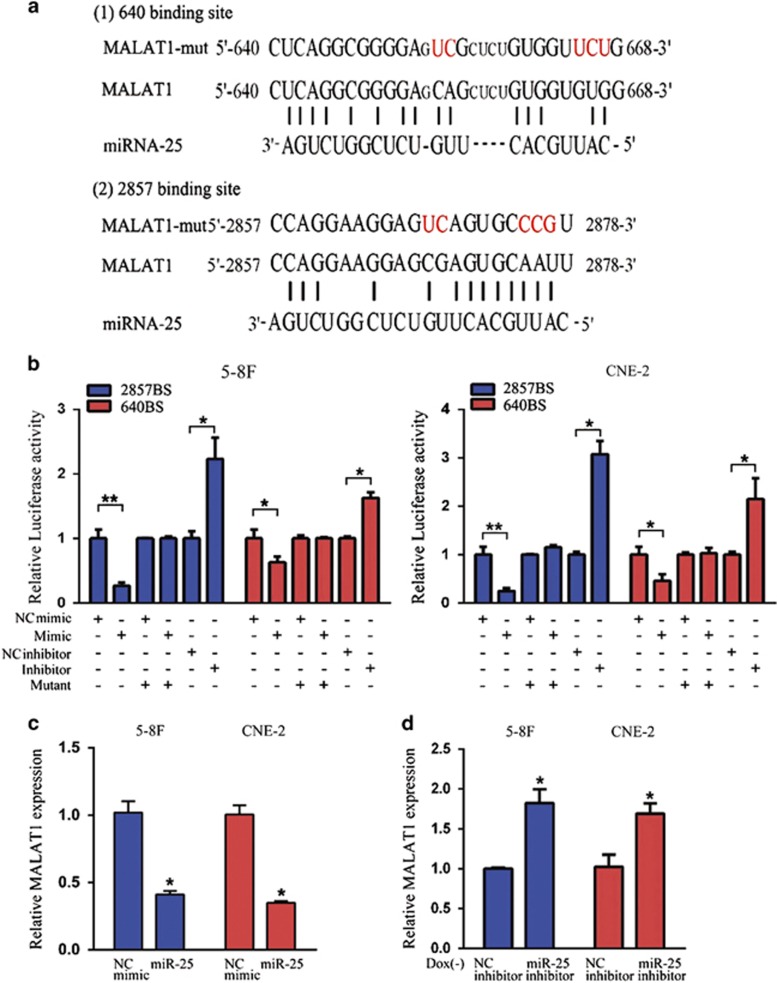
miR-25 reduced the MALAT1 level. (**a**) Schematic constructions of the wild type and mutant pMIR-REPORT-MALAT1 miRNA expression vectors used in luciferase reporter assays. The five altered nucleotides in the mutant binding site are colored in red. (**b**) The relative luciferase activities in the 5-8 F and CNE-2 cells after transfection with the pMIR-REPORT-MALAT1 reporter and miR-25 mimic, NC mimic, miR-25 inhibitor or NC inhibitor. (**c**) QRT-PCR analysis of MALAT1 expression in 5-8 F and CNE-2 cells that were transfected with miR-25 mimic (50 nM) or NC mimic. (**d**) QRT-PCR analysis of MALAT1 expression in 5-8 F and CNE-2 Tet-Off-inducible RBM24-stable cells that were transfected with miR-25 inhibitor (100 nM) or NC inhibitor after the removal of doxycycline for 24 h. All data are shown as the mean±S.E.M. of three independent experiments (*P<0.05 and **P<0.01, Student's *t-*test)

To gain insights into the mechanisms underlying the tumor suppressive function of miR-25, we next identified its target genes in NPC. We have recently reported that miR-103 and miR-107 degrade the lncRNA NKILA in breast cancer cells.^34^ Based on these results, we used a miRNA-lncRNA interaction analysis program, starBase v2.0, which employs a database containing a large set of Ago and RBP binding sites derived from all available CLIP-Seq experimental techniques, to screen for potential lncRNAs targeted by miR-25. The results showed that 33 lncRNAs were possible targets of miR-25 (Supplementary Table S2). Notably, the lncRNA MALAT1, an oncogenic lncRNA that is overexpressed in various human cancers, was the most common lncRNA target of miR-25 in all cancers according to the analysis results. Next, we used RNA hybrid programs to identify two putative binding sites for miR-25 in the MALAT1 sequence (NR_002819.2) at positions 640 and 2857 (Figure 5a and Supplementary Figure S3). To validate the predicted miR-25-binding sites, luciferase reporter assay was performed, which showed that miR-25 overexpression resulted in a significant decrease in luciferase activity, whereas the opposite effect was observed when this miRNA was inhibited (Figure 5b). Moreover, mutation of the predicted miR-25-binding sites abolished this effect (Figure 5b). Furthermore, we found that MALAT1 expression was significantly reduced by transfection with miR-25 mimic compared with negative control miRNA mimic (NC mimic) (Figure 5c). In contrast, miR-25 inhibitor significantly restored the MALAT1 RNA level in RBM24-induced cells (Figure 5d). Moreover, we also observed that the increase or decrease in the expression of lncRNA XIST was consistent for the expression of MALAT1 when suppressing or overexpressing miR-25 expression in the 5-8 F and CNE-2 cells (Supplementary Figure S4). Altogether, these results suggest that MALAT1 is a target of miR-25.

### RBM24 inhibits MALAT1 expression by upregulating the miR-25 level

**Figure 6:**
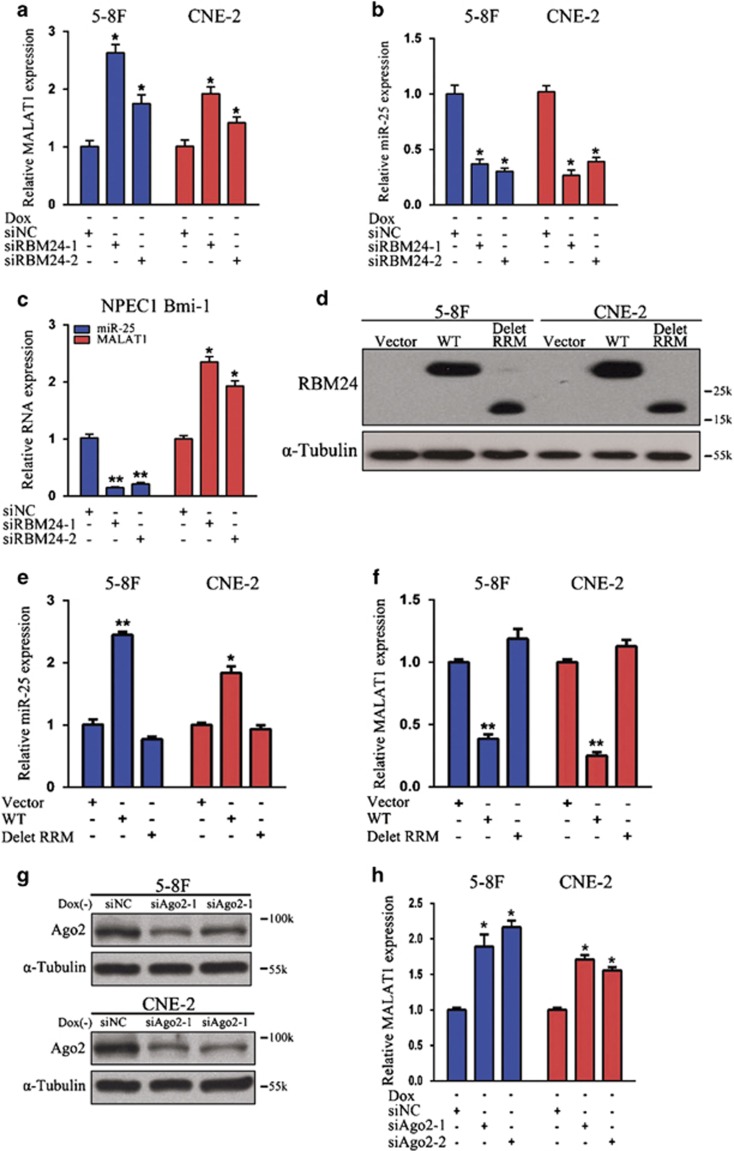
RBM24 reduced the MALAT1 level by upregulating miR-25. (**a** and **b**) QRT-PCR, revealing MALAT1 and miR-25 expression following RBM24 knockdown in 5-8 F and CNE-2 Tet-Off-inducible RBM24-stable cells after the removal of doxycycline for 24 h. (**c**) QRT-PCR analysis of miR-25 and MALAT1 expression in NPEC1 Bmi-1 cells that were transiently transfected with siRNAs (50 nM) against *RBM24* or scramble siRNAs as controls. (**d**) Western blotting analysis of RBM24 expression in 5-8 F and CNE-2 cells that were transfected with RBM24 overexpression vector, RRM deletion vector or control vector for 48 h. (**e** and **f**) QRT-PCR analysis of miR-25 and MALAT1 expression in 5-8 F and CNE-2 cells treated as in (**d**). (**g**) Western blotting analysis, verifying the knockdown of Ago2 in 5-8 F and CNE-2 Tet-Off-inducible RBM24-stable cells after the removal of doxycycline for 24 h. (**h**) QRT-PCR analysis of MALAT1 expression in 5-8 F and CNE-2 Tet-Off-inducible RBM24-stable cells treated as in (**g**). All data are shown as the mean±S.E.M. of three independent experiments (*P<0.05 and **P<0.01, Student's *t*-test)

To assess whether and how RBM24 expression inhibits MALAT1 expression, we found that MALAT1 expression was significantly increased (Figure 6a), while miR-25 expression was significantly decreased (Figure 6b), in RBM24-induced cells following the knock down of RBM24 compared with that in the respective control cells. Moreover, the silencing of endogenous RBM24 in NPEC1 Bmi-1 cell line resulted in a significant reduction in the miR-25 expression and increase in the MALAT1 expression compared with scramble siRNAs (Figure 6c). Next, we compared miR-25 and MALAT1 expression following transfection of NPC cells with an RBM24 overexpression vector *versus* a control vector. As shown in Figures 6d–f, RBM24 overexpression resulted in a significant increase in the miR-25 expression and reduction in the MALAT1 RNA level compared with control vector transfection in 5-8 F and CNE-2 cells, whereas deletion of the RRM of RBM24 abolished the effects of miR-25 upregulation and MALAT1 inhibition. To determine whether the reduction in MALAT1 expression by RBM24 was mediated by miRNA, we knocked down the expression of Ago2 in RBM24-induced cells and subsequently observed an increase in MALAT1 RNA expression (Figures 6g and h). This increase may have been due to the inhibition of miR-25 function in the RBM24-induced cells following Ago2 knockdown. Taken together, these finding demonstrate that RBM24 inhibits the expression of MALAT1 through upregulation of the expression of miR-25, which directly targets MALAT1 for degradation.

### RBM24 suppresses NPC tumor formation and metastasis

**Figure 7:**
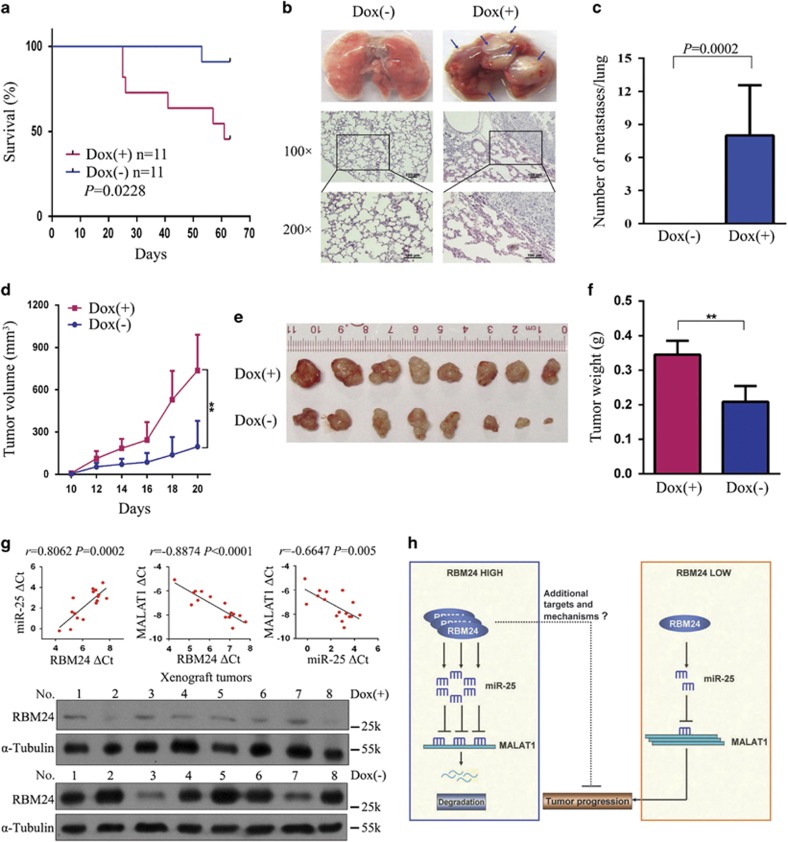
RBM24 suppresses the growth and metastasis of NPC tumors *in vivo*. (**a**) Kaplan–Meier survival curves of overall survival duration based on RBM24 expression in the nude mice that were treated with or without doxycycline. (**b**) Upper: representative images of macroscopic lung metastases; the arrowheads indicate the metastatic nodes. Lower: representative H&E staining of the lung metastatic tumors is shown. (**c**) Quantification of the average number of metastatic nodes on the surfaces of the lungs (the data are presented as the mean±S.D., Student's *t*-test). (**d**) The growth curve indicates that the growth of 5-8 F Tet-Off-inducible RBM24-stable cells was suppressed *in vivo* after the removal of doxycycline (*n*=8 per group, the data are presented as the mean±S.D., **P<0.01, Student's *t*-test). (**e**) Representative images of xenograft tumors in nude mice after treatment with or without doxycycline. (**f**) The terminal tumor weight was measured for each group. Representative tumor sizes are shown (the data are presented as the mean±SD, **P<0.01, Student's *t*-test). (**g**) Upper: Pearson correlation analysis of the RBM24, miR-25 and MALAT1 RNA levels in the freshly xenografted tumors, as represented by Pearson *R* scores (*n*=16, *R*<0 denotes negative correlation). Lower: the relative levels of RBM24 protein in the freshly xenografted tumors, as determined by immunoblotting. (**h**) Model of RBM24-induced modulation of the stability and tumorigenesis of MALAT1 by miR-25

To investigate whether RBM24 is crucial for the regulation of NPC tumor growth and metastasis *in vivo* and to determine the survival period of tumor-bearing mice, 1 × 10^6^ highly metastatic 5-8 F Tet-Off-inducible RBM24 stable cells were injected into nude mice. Although the palpable tumors formed from the induced cells and control cells appeared at similar times and grew at comparable rates, the Kaplan–Meier survival curves showed a greater survival rate when RBM24 was induced (Figure 7a). When the mice were killed at 9 weeks after injection, we observed pulmonary metastases in all mice from the control group (Figure 7b). In contrast, there were no obvious pulmonary metastatic nodes in the RBM24-induced mice (Figure 7c). The pulmonary metastatic tumors were examined using hematoxylin and eosin staining (Figure 7b). Because there was no significant difference in tumor growth when the mice were inoculated with a large amount of cancer cells, we assessed the effects of RBM24 on NPC cell growth *in vivo* with a reduction in the number of inoculated cells to 5 × 10^4^ cells. The tumor growth curves derived from the xenograft experiments indicated that induction of RBM24 expression impeded the growth of NPC cells in nude mice (Figure 7d). The final tumor weights and photographs of the isolated tumors are shown in Figures 7e and f. To further investigate the correlations between RBM24, miR-25 and MALAT1 expression *in vivo*, we performed qRT-PCR to determine the RBM24, miR-25 and MALAT1 RNA levels in the xenografted tumors. As shown in Figure 7g, the *RBM24* mRNA level was positively associated with miR-25 expression (*P*=0.0002, *r*=0.8026) and negatively associated with MALAT1 expression (*P*<0.0001, *r*=−0.8874). We also observed a negative association between miR-25 and MALAT1 expression (*P*=0.005, *r*=−0.6647). In addition, similar results were obtained from correlation analysis among the expression levels of RBM24, miR-25, and MALAT1 in the primary NPC fresh tumor tissues (Supplementary Figure S5). Taken together, these results suggest that the suppression of tumorigenicity and invasiveness by RBM24 depends, to a certain extent, on upregulation of the level of miR-25, which in turn targets MALAT1 for degradation.

## Discussion

RBPs are known to play key roles in co-transcriptional and post-transcriptional regulation, including alternative splicing, alternative polyadenylation, transcript stability, translation and other processing steps.^35, 36, 37^ The interactions of RBPs and their RNA targets are often mediated by one or more RNA-binding domains, such as the RRM and hnRNPK-homology domains. The precise regulation of RNA metabolism is fundamental to the generation of biological complexity in both disease and normal states.^38, 39^ Recently, a great deal of attention has been paid to the essential roles played by miRNAs and RBPs, which interact with the *cis*-regulatory elements of transcripts to regulate gene expression. Accumulating evidence indicates that the dysregulation of RBPs leads to alterations in cellular processes and normal development and results in the development of various disorders.^33, 40^

In the present study, we conducted *in vitro* and *in vivo* assays, demonstrating that RBM24 had a strong tumor suppressive potential in NPC cells. RBM24 has been shown to alter the mRNA stabilities of p21 and p63 in different cancer cell lines.^30, 31^ Many recent studies have found that the dysregulation of miRNAs in NPC is related to disease prognosis and clinical outcome.^16, 17, 18, 19^ We compared the miRNA expression profiles of three Tet-Off-inducible RBM24-stable cell lines and found that miR-25 was the most significantly upregulated miRNA in RBM24-induced cells. This miRNA is hosted by the minichromosome maintenance protein-7 gene and is transcribed as part of the miR-106b~25 cluster.^41^ Previous reports have demonstrated that miR-25 is overexpressed in a number of cancers, including gastric, lung, liver and ovarian cancers.^42, 43, 44, 45^ However, it has also been shown to be downregulated in colon cancer compared with its expression in matched non-tumor tissues, whereas the restoration of its expression inhibits cell proliferation and migration.^46^ Similarly, an elevated miR-25 level in NPC cells has been associated with the significant inhibition of cell proliferation, migration and invasion.

In recent years, dysregulated lncRNAs have been shown to function as tumor suppressors or oncogenes.^13, 14, 15^ However, the precise mechanisms and functions of most of these lncRNAs remain largely unknown. In this study, we searched a database of miRNA-lncRNA interactions using starBase v2.0 ^47^ and found that miR-25 bound to numerous lncRNAs. Interestingly, a well-known oncogenic lncRNA, MALAT1, was the most common among 33 lncRNAs that were bound by miR-25 in all cancers. Thus, we conducted experiments to confirm that miR-25 can bind to and inhibit the expression of MALAT1 in NPC cells. Furthermore, we observed that the knockdown of Ago2 expression, which is the only component of the human RNA-induced silencing complex to have slicer activity,^48^ blocked the inhibitory effects of miR-25 on MALAT1-regulated processes. Functional assays revealed that the knockdown of MALAT1 expression in stable cells without induction of RBM24 expression had similar or even stronger inhibitory effects on cellular proliferation, motility and invasion compared with the induction of RBM24 expression, and these inhibitory effects were negatively correlated with MALAT1 expression in various cells (Supplementary Figure S6). On the basis of our findings, we propose a model in which RBM24 plays a role as a tumor suppressor through the upregulation of miR-25, which directly targets MALAT1 for degradation (Figure 7h). Other recent studies have reported that miR-9, miR-101 and miR-217 target MALAT1 for degradation in other human cancer cell lines.^49, 50^ This evidence also supports our finding that miR-25 downregulates MALAT1 expression in NPC.

In summary, we identified RBM24 as a novel tumor suppressor in NPC. RBM24 exerts its inhibitory effects, at least in part, by upregulating the level of miR-25, which in turn targets MALAT1 for degradation in an Ago2-dependent manner. Further studies of RBM24 expression in patient-derived tumor tissues will help to determine its clinical significance in NPC diagnosis and treatment. In addition, identifying whether RBM24 plays a role as a tumor suppressor in other human malignancies will be of great interest.

## Materials and Methods

### Clinical specimens and cell lines

Primary NPC fresh tumor tissues and fresh non-cancer nasopharyngitis tissues were acquired from the Tumor Tissue Bank of Sun Yat-Sen University Cancer Center, and the detailed relevant information has been described previously.^32^ This study was approved by the Institute Research Medical Ethics Committee of Sun Yat-Sen University Cancer Center, and written informed consent was obtained from all patients. NPC cell lines were maintained in RPMI 1640 medium (Life Technologies, Carlsbad, CA, USA) supplemented with 10% fetal bovine serum (FBS) in a humidified 5% CO_2_ incubator at 37 °C. Primary NPEC1 cultures and immortalized NPEC1 cells induced by Bmi-1 were established as described previously^51^ and grown in keratinocyte/serum-free medium (Invitrogen, Carlsbad, CA, USA). Tet-Off-inducible advanced cell lines were maintained in RPMI 1640 medium supplemented with 10% Tet-System Approved FBS (Clontech, Mountain View, CA, USA) with both G418 (400 *μ*g/ml) and doxycycline (200 ng/ml). Tet-Off-inducible RBM24 stable cells were maintained in RPMI 1640 medium supplemented with 10% Tet-System Approved FBS (Clontech) with G418 (400 *μ*g/ml), puromycin (0.5 *μ*g/ml) and doxycycline (200 ng/ml).

### Antibodies and reagents

Rabbit anti-RBM24 was obtained from Sigma (St. Louis, MO, USA) (SAB1103678); mouse anti-Ago2 was purchased from Abcam (Cambridge, MA, USA) (ab57113); mouse anti-*α* tubulin was purchased from Abcam (ab7291). Goat anti-mouse and goat anti-rabbit peroxidase-conjugated secondary antibodies were obtained from Amersham Pharmacia Biotech (Piscataway, NJ, USA). Doxycycline was obtained from Clontech (631311). The siRNAs, miR-25 mimic, miR-25 inhibitor and scrambled negative control were purchased from Ribobio Co (Guangzhou, China). The siRNAs sequences are listed in Supplementary Table S3.

### RNA extraction, cDNA synthesis and qPCR

Total RNA was extracted using TRIzol reagent (Invitrogen). First-strand cDNA was synthesized from 1 *μ*g total RNA using a GoScript Reverse Transcription System (Promega, Madison, WI, USA) and was then used for qPCR analysis. For analysis of miRNA expression, RNA was reverse transcribed using Bulge-Loop miRNA-specific RT primers (Ribobio). qPCR was carried out using SYBR Green PCR master mix (Bio-Rad, Hercules, CA, USA) and a LightCycler 480 II System (Roche, Indianapolis, IN, USA). The snRNA U6 or GAPDH was used as an internal control to normalize the expression levels of the different genes. The primers used for amplification of the indicated genes are listed in Supplementary Table S4.

### Western blot analysis

Total cell and tissues lysates were prepared in RIPA buffer (100 mM Tris-HCl, 300 mM NaCl, 2% NP40 and 0.5% sodium deoxycholate) supplemented with Protease Inhibitor Cocktail (Roche). Protein concentrations were assayed using a BCA Protein Assay Kit (Pierce Biotechnology, Rockford, IL, USA). Identical quantities of proteins were separated by SDS-PAGE and transferred to polyvinylidene difluoride membranes (Millipore, Billerica, MA, USA). After blocking with 5% skim milk or BSA in Tris-buffered saline/Tween-20 (TBST), the membranes were incubated with the primary antibodies overnight at 4 °C and then with the respective horseradish peroxidase-conjugated secondary antibodies at room temperature for 2 h. Protein bands were visualized using a chemiluminescence kit (Pierce, Billerica, MA, USA). *α*-Tubulin was used as a loading control for the western blots.

### Cell proliferation and foci formation assays

Cell proliferation rates were determined with a Cell Counting Kit-8 (CCK-8), which is used to perform a sensitive colorimetric assay of viable cells, according to the manufacturer's protocol (Dojindo Laboratories, Kumamoto, Japan). Briefly, 1 × 10^3^ cells/well were seeded in triplicate in 96-well culture plates. Parallel culture plates were harvested at various time points post-seeding, and 10 *μ*l CCK-8 solution was added to each well. The solution was incubated with the cells for an additional hour, and optical density (OD) was then measured at 450 nm. For cell foci formation assay, cells were seeded in triplicate in six-well culture plates at a density of 1000 cells/well. The culture medium was subsequently changed every 3 days. After 2 weeks, the resulting colonies were fixed with methanol and stained with 0.1% crystal violet. Colonies that contained greater than 50 cells were counted.

### Anchorage-independent growth assays

Six-well plates were covered with a layer of 0.6% agar in medium supplemented with 20% FBS. Cells were prepared in 0.3% agar and seeded in triplicate at three different dilutions, ranging from 1 × 10^3^ to 5 × 10^5^. The plates were incubated at 37 °C in a humid atmosphere of 5% CO_2_ for 4 weeks. Each experiment was repeated at least three times. Colonies were photographed between 18 and 24 days at an original magnification of × 200 under phase contrast.

### Migration and invasion assays

Migration and invasion assays were performed using cell culture inserts with 8-μm pore transparent polyethylene terephthalate filters (Becton Dickinson, Bedford, MA, USA) coated with (for invasion assays) or without (for migration assays) Matrigel. A total of 3 × 10^4^ cells suspended in 200 *μ*l serum-free culture medium were added to each insert, and 500 *μ*l culture medium containing 10% FBS was added to the bottom chamber. After incubation for 24 h at 37 °C, the cells on the upper filter were removed, and those that migrated or invaded the lower surface of the membrane were fixed in methanol and stained with crystal violet. Five optical fields ( × 100 magnification) for each filter with triplicate inserts were randomly selected to calculate the number of migrated or invaded cells.

### Luciferase reporter assay

Two putative miR-25 binding sites in the MALAT1 RNA were cloned downstream of the cytomegalovirus (CMV) promoter in a pMIR-REPORT vector (Ambion, Carlsbad, CA, USA). The MALAT1-binding sites at positions 640 and 2857 containing partial MALAT1 sequences were located at 484–878 nt and 2628–3102 nt, respectively. Luciferase activity was measured using a Dual-Luciferase Reporter Assay System (Promega) according to the manufacturer's instructions. Briefly, pMIR-REPORT-MALAT1 or pMIR-REPORT-MALAT1-mut was cotransfected with miR-25 mimic, inhibitor, or the corresponding negative control into 5-8 F and CNE-2 cells by Lipofectamine-mediated gene transfer. Relative luciferase activity was normalized to Renilla luciferase activity at 48 h after transfection.

### Microarray analysis

Tet-Off-inducible RBM24 stable cells were cultured with doxycycline. For miRNA array analysis, doxycycline was removed from the culture medium after 48 h, and total RNA was isolated as described previously.^52^ Next, a microarray containing 873 miRNA probes was employed according to previously described miRNA probe design, RNA labeling and microarray hybridization methods.^53, 54^ Briefly, 2.5 *μ*g total RNA was labeled with pCp-DY647 (Dharmacon, Lafayette, CO, USA). After hybridization, the arrays were scanned with a LuxScan 10 K Microarray Scanner (CapitalBio, Beijing, China), and the resulting images were analyzed with GenePix Pro 6.0 software (Axon Instruments, Foster City, CA, USA).

### *In vivo* experiments

Female 4- to 5-week-old athymic mice were purchased (BALB/c nu/nu; Guangdong Medical Laboratory Animal Center, Guangzhou, China) and were maintained under a specific pathogen-free environment. All animal experiments were approved by the Institutional Animal Care and Use Committee of Sun Yat-sen University Cancer Center. For the tumor xenograft experiments, tumor cells (5 × 10^4^ or 1 × 10^6^ cells/tumor in 100 *μ*l of serum-free culture medium) were suspended in 200 *μ*l RPMI 1640 complete culture medium with 25% Matrigel (BD Biosciences, Bedford, MA, USA) and inoculated subcutaneously into the right flanks of the nude mice. The mice were monitored daily for palpable tumor formation, and tumors were measured using a Vernier caliper, weighed and photographed. Tumor width (*W*) and length (*L*) were measured every 2 days. RBM24 expression was repressed by the addition of doxycycline (1 g/l) to the drinking water until the mice were killed at 3 (5 × 10^4^ cells/tumor, *n*=16) or 9 weeks (1 × 10^6^ cells/tumor, *n*=22) after inoculation. Then, the tumors were isolated and weighed. Tumor volumes were calculated using the formula *V*=1/2 (*L* × *W*^2^).

### Accession numbers

The Gene Expression Omnibus database accession number for the miRNA array data reported in this paper is GSE66878.

### Statistical analysis

All *in vitro* experiments were repeated at least three times unless stated otherwise. Differences among the groups and treatments were determined by Student's *t*-test unless stated otherwise. Kaplan–Meier survival analyses were performed to compare the survival times between the RBM24-induced and non-induced mice, and the log-rank test was used to generate *P-*values. The differences were considered significant at a *P*<0.05.

## Abbreviations

RBPs: RNA-binding proteins
RBM24: RNA-binding protein 24
RRM: RNA recognition motif
Dox: Doxycycline
WT: Wild type
Ago2: Argonaute2
lncRNAs: long noncoding RNAs
MALAT1: metastasis associated in lung adenocarcinoma transcript 1
NPC: nasopharyngeal carcinoma

## References

[bib1] Hong RL, Ting LL, Ko JY, Hsu MM, Sheen TS, Lou PJ et al. Induction chemotherapy with mitomycin, epirubicin, cisplatin, fluorouracil, and leucovorin followed by radiotherapy in the treatment of locoregionally advanced nasopharyngeal carcinoma. J Clin Oncol 2001; 19: 4305–4313. 11731513 10.1200/JCO.2001.19.23.4305

[bib2] Lin JC, Jan JS, Hsu CY, Liang WM, Jiang RS, Wang WY. Phase III study of concurrent chemoradiotherapy versus radiotherapy alone for advanced nasopharyngeal carcinoma: positive effect on overall and progression-free survival. J Clin Oncol 2003; 21: 631–637. 12586799 10.1200/JCO.2003.06.158

[bib3] Liu N, Chen NY, Cui RX, Li WF, Li Y, Wei RR et al. Prognostic value of a microRNA signature in nasopharyngeal carcinoma: a microRNA expression analysis. Lancet Oncol 2012; 13: 633–641. 22560814 10.1016/S1470-2045(12)70102-X

[bib4] Wang LJ, Chou YF, Chen PR, Su B, Hsu YC, Chang CH et al. Differential miRNA expression in repeated recurrence of nasopharyngeal carcinoma. Cancer Lett 2014; 344: 188–194. 24183849 10.1016/j.canlet.2013.10.023

[bib5] Sengupta S, den Boon JA, Chen IH, Newton MA, Stanhope SA, Cheng YJ et al. MicroRNA 29c is down-regulated in nasopharyngeal carcinomas, up-regulating mRNAs encoding extracellular matrix proteins. Proc Natl Acad Sci USA 2008; 105: 5874–5878. 18390668 10.1073/pnas.0801130105 PMC2311339

[bib6] Zhang JX, Qian D, Wang FW, Liao DZ, Wei JH, Tong ZT et al. MicroRNA-29c enhances the sensitivities of human nasopharyngeal carcinoma to cisplatin-based chemotherapy and radiotherapy. Cancer Lett 2013; 329: 91–98. 23142283 10.1016/j.canlet.2012.10.033

[bib7] Lu J, Xu X, Liu X, Peng Y, Zhang B, Wang L et al. Predictive value of miR-9 as a potential biomarker for nasopharyngeal carcinoma metastasis. Br J Cancer 2014; 110: 392–398. 24327016 10.1038/bjc.2013.751 PMC3899774

[bib8] Lu J, Luo H, Liu X, Peng Y, Zhang B, Wang L et al. miR-9 targets CXCR4 and functions as a potential tumor suppressor in nasopharyngeal carcinoma. Carcinogenesis 2014; 35: 554–563. 24170200 10.1093/carcin/bgt354

[bib9] Gao F, Zhao ZL, Zhao WT, Fan QR, Wang SC, Li J et al. miR-9 modulates the expression of interferon-regulated genes and MHC class I molecules in human nasopharyngeal carcinoma cells. Biochem Biophys Res Commun 2013; 431: 610–616. 23291181 10.1016/j.bbrc.2012.12.097

[bib10] Yu L, Lu J, Zhang B, Liu X, Wang L, Li SY et al. miR-26a inhibits invasion and metastasis of nasopharyngeal cancer by targeting EZH2. Oncol Lett 2013; 5: 1223–1228. 23599767 10.3892/ol.2013.1173 PMC3629195

[bib11] Alajez NM, Shi W, Hui AB, Bruce J, Lenarduzzi M, Ito E et al. Enhancer of Zeste homolog 2 (EZH2) is overexpressed in recurrent nasopharyngeal carcinoma and is regulated by miR-26a, miR-101, and miR-98. Cell Death Dis 2010; 1: e85. 21368858 10.1038/cddis.2010.64 PMC3035896

[bib12] Lu J, He ML, Wang L, Chen Y, Liu X, Dong Q et al. MiR-26a inhibits cell growth and tumorigenesis of nasopharyngeal carcinoma through repression of EZH2. Cancer Res 2011; 71: 225–233. 21199804 10.1158/0008-5472.CAN-10-1850

[bib13] Schmitt AM, Chang HY. Gene regulation: long RNAs wire up cancer growth. Nature 2013; 500: 536–537. 23945584 10.1038/nature12548 PMC5332550

[bib14] Prensner JR, Chinnaiyan AM. The emergence of lncRNAs in cancer biology. Cancer Discov 2011; 1: 391–407. 22096659 10.1158/2159-8290.CD-11-0209 PMC3215093

[bib15] Mitra SA, Mitra AP, Triche TJ. A central role for long non-coding RNA in cancer. Front Genet 2012; 3: 17. 22363342 10.3389/fgene.2012.00017 PMC3279698

[bib16] Yang QQ, Deng YF. Genome-wide analysis of long non-coding RNA in primary nasopharyngeal carcinoma by microarray. Histopathology 2015; 66: 1022–1030. 25406670 10.1111/his.12616

[bib17] Gong Z, Zhang S, Zeng Z, Wu H, Yang Q, Xiong F et al. LOC401317, a p53-regulated long non-coding RNA, inhibits cell proliferation and induces apoptosis in the nasopharyngeal carcinoma cell line HNE2. PLoS One 2014; 9: e110674. 25422887 10.1371/journal.pone.0110674 PMC4244030

[bib18] Gao W, Chan JY, Wong TS. Differential expression of long noncoding RNA in primary and recurrent nasopharyngeal carcinoma. Biomed Res Int 2014; 2014: 404567. 24822202 10.1155/2014/404567 PMC4009106

[bib19] Nie Y, Liu X, Qu S, Song E, Zou H, Gong C. Long non-coding RNA HOTAIR is an independent prognostic marker for nasopharyngeal carcinoma progression and survival. Cancer Sci 2013; 104: 458–464. 23281836 10.1111/cas.12092 PMC7657223

[bib20] Ren S, Liu Y, Xu W, Sun Y, Lu J, Wang F et al. Long noncoding RNA MALAT-1 is a new potential therapeutic target for castration resistant prostate cancer. J Urol 2013; 190: 2278–2287. 23845456 10.1016/j.juro.2013.07.001

[bib21] Fan Y, Shen B, Tan M, Mu X, Qin Y, Zhang F et al. TGF-beta-induced upregulation of malat1 promotes bladder cancer metastasis by associating with suz12. Clin Cancer Res 2014; 20: 1531–1541. 24449823 10.1158/1078-0432.CCR-13-1455

[bib22] Zhao Z, Chen C, Liu Y, Wu C. 17beta-Estradiol treatment inhibits breast cell proliferation, migration and invasion by decreasing MALAT-1 RNA level. Biochem Biophys Res Commun 2014; 445: 388–393. 24525122 10.1016/j.bbrc.2014.02.006

[bib23] Xie L, Hu Z, Wang X, Li Z. Expression of long noncoding RNA MALAT1 gene in human nasopharyngeal carcinoma cell lines and its biological significance. Nan Fang Yi Ke Da Xue Xue Bao 2013; 33: 692–697. 23688988

[bib24] Gutschner T, Hammerle M, Diederichs S. MALAT1 – a paradigm for long noncoding RNA function in cancer. J Mol Med (Berl) 2013; 91: 791–801. 23529762 10.1007/s00109-013-1028-y

[bib25] Terami H, Hidaka K, Shirai M, Narumiya H, Kuroyanagi T, Arai Y et al. Efficient capture of cardiogenesis-associated genes expressed in ES cells. Biochem Biophys Res Commun 2007; 355: 47–53. 17286962 10.1016/j.bbrc.2007.01.109

[bib26] Jin D, Hidaka K, Shirai M, Morisaki T. RNA-binding motif protein 24 regulates myogenin expression and promotes myogenic differentiation. Genes Cells 2010; 15: 1158–1167. 20977548 10.1111/j.1365-2443.2010.01446.x

[bib27] Poon KL, Tan KT, Wei YY, Ng CP, Colman A, Korzh V et al. RNA-binding protein RBM24 is required for sarcomere assembly and heart contractility. Cardiovasc Res 2012; 94: 418–427. 22345307 10.1093/cvr/cvs095

[bib28] Grifone R, Xie X, Bourgeois A, Saquet A, Duprez D, Shi DL. The RNA-binding protein Rbm24 is transiently expressed in myoblasts and is required for myogenic differentiation during vertebrate development. Mech Dev 2014; 134: 1–15. 25217815 10.1016/j.mod.2014.08.003

[bib29] Yang J, Hung LH, Licht T, Kostin S, Looso M, Khrameeva E et al. RBM24 is a major regulator of muscle-specific alternative splicing. Dev Cell 2014; 31: 87–99. 25313962 10.1016/j.devcel.2014.08.025

[bib30] Jiang Y, Zhang M, Qian Y, Xu E, Zhang J, Chen X. Rbm24, an RNA-binding protein and a target of p53, regulates p21 expression via mRNA stability. J Biol Chem 2014; 289: 3164–3175. 24356969 10.1074/jbc.M113.524413 PMC3916521

[bib31] Xu E, Zhang J, Zhang M, Jiang Y, Cho SJ, Chen X. RNA-binding protein RBM24 regulates p63 expression via mRNA stability. Mol Cancer Res 2014; 12: 359–369. 24375645 10.1158/1541-7786.MCR-13-0526 PMC3962715

[bib32] Yuan L, Liu ZH, Lin ZR, Xu LH, Zhong Q, Zeng MS. Recurrent FGFR3-TACC3 fusion gene in nasopharyngeal carcinoma. Cancer Biol Ther 2014; 15: 1613–1621. 25535896 10.4161/15384047.2014.961874 PMC4622012

[bib33] van Kouwenhove M, Kedde M, Agami R. MicroRNA regulation by RNA-binding proteins and its implications for cancer. Nat Rev Cancer 2011; 11: 644–656. 21822212 10.1038/nrc3107

[bib34] Liu B, Sun L, Liu Q, Gong C, Yao Y, Lv X et al. A cytoplasmic NF-kappaB interacting long noncoding RNA blocks IkappaB phosphorylation and suppresses breast cancer metastasis. Cancer Cell 2015; 27: 370–381. 25759022 10.1016/j.ccell.2015.02.004

[bib35] Moore MJ, Proudfoot NJ. Pre-mRNA processing reaches back to transcription and ahead to translation. Cell 2009; 136: 688–700. 19239889 10.1016/j.cell.2009.02.001

[bib36] Glisovic T, Bachorik JL, Yong J, Dreyfuss G. RNA-binding proteins and post-transcriptional gene regulation. FEBS Lett 2008; 582: 1977–1986. 18342629 10.1016/j.febslet.2008.03.004 PMC2858862

[bib37] Keene JD. RNA regulons: coordination of post-transcriptional events. Nat Rev Genet 2007; 8: 533–543. 17572691 10.1038/nrg2111

[bib38] Sharp PA. The centrality of RNA. Cell 2009; 136: 577–580. 19239877 10.1016/j.cell.2009.02.007

[bib39] Licatalosi DD, Darnell RB. RNA processing and its regulation: global insights into biological networks. Nat Rev Genet 11: 75–87. 20019688 10.1038/nrg2673 PMC3229837

[bib40] Ciafre SA, Galardi S. microRNAs and RNA-binding proteins: a complex network of interactions and reciprocal regulations in cancer. RNA Biol 2013; 10: 935–942. 23696003 10.4161/rna.24641 PMC4111733

[bib41] Kan T, Sato F, Ito T, Matsumura N, David S, Cheng Y et al. The miR-106b-25 polycistron, activated by genomic amplification, functions as an oncogene by suppressing p21 and Bim. Gastroenterology 2009; 136: 1689–1700. 19422085 10.1053/j.gastro.2009.02.002 PMC2887605

[bib42] Li BS, Zuo QF, Zhao YL, Xiao B, Zhuang Y, Mao XH et al. MicroRNA-25 promotes gastric cancer migration, invasion and proliferation by directly targeting transducer of ERBB2, 1 and correlates with poor survival. Oncogene 2015; 34: 2556–2565. 25043310 10.1038/onc.2014.214

[bib43] Xu FX, Su YL, Zhang H, Kong JY, Yu H, Qian BY. Prognostic implications for high expression of MiR-25 in lung adenocarcinomas of female non-smokers. Asian Pac J Cancer Prev 2014; 15: 1197–1203. 24606441 10.7314/apjcp.2014.15.3.1197

[bib44] Su ZX, Zhao J, Rong ZH, Geng WM, Wu YG, Qin CK. Upregulation of microRNA-25 associates with prognosis in hepatocellular carcinoma. Diagn Pathol 2014; 9: 47. 24593846 10.1186/1746-1596-9-47 PMC4016611

[bib45] Wang X, Meng X, Li H, Liu W, Shen S, Gao Z. MicroRNA-25 expression level is an independent prognostic factor in epithelial ovarian cancer. Clin Transl Oncol 2014; 16: 954–958. 24696291 10.1007/s12094-014-1178-6

[bib46] Li Q, Zou C, Zou C, Han Z, Xiao H, Wei H et al. MicroRNA-25 functions as a potential tumor suppressor in colon cancer by targeting Smad7. Cancer Lett 2013; 335: 168–174. 23435373 10.1016/j.canlet.2013.02.029

[bib47] Li JH, Liu S, Zhou H, Qu LH, Yang JH. starBase v2.0: decoding miRNA-ceRNA, miRNA-ncRNA and protein-RNA interaction networks from large-scale CLIP-Seq data. Nucleic Acids Res 2014; 42: D92–D97. 24297251 10.1093/nar/gkt1248 PMC3964941

[bib48] Liu J, Carmell MA, Rivas FV, Marsden CG, Thomson JM, Song JJ et al. Argonaute2 is the catalytic engine of mammalian RNAi. Science 2004; 305: 1437–1441. 15284456 10.1126/science.1102513

[bib49] Leucci E, Patella F, Waage J, Holmstrom K, Lindow M, Porse B et al. microRNA-9 targets the long non-coding RNA MALAT1 for degradation in the nucleus. Sci Rep 2013; 3: 2535. 23985560 10.1038/srep02535 PMC3756333

[bib50] Wang X, Li M, Wang Z, Han S, Tang X, Ge Y et al. Silencing of long noncoding RNA MALAT1 by miR-101 and miR-217 inhibits proliferation, migration, and invasion of esophageal squamous cell carcinoma cells. J Biol Chem 2015; 290: 3925–3935. 25538231 10.1074/jbc.M114.596866 PMC4326802

[bib51] Song LB, Zeng MS, Liao WT, Zhang L, Mo HY, Liu WL et al. Bmi-1 is a novel molecular marker of nasopharyngeal carcinoma progression and immortalizes primary human nasopharyngeal epithelial cells. Cancer Res 2006; 66: 6225–6232. 16778197 10.1158/0008-5472.CAN-06-0094

[bib52] Song LB, Li J, Liao WT, Feng Y, Yu CP, Hu LJ et al. The polycomb group protein Bmi-1 represses the tumor suppressor PTEN and induces epithelial-mesenchymal transition in human nasopharyngeal epithelial cells. J Clin Invest 2009; 119: 3626–3636. 19884659 10.1172/JCI39374 PMC2786794

[bib53] Wang HY, Luo M, Tereshchenko IV, Frikker DM, Cui X, Li JY et al. A genotyping system capable of simultaneously analyzing >1000 single nucleotide polymorphisms in a haploid genome. Genome Res 2005; 15: 276–283. 15687291 10.1101/gr.2885205 PMC546529

[bib54] Wang H, Ach RA, Curry B. Direct and sensitive miRNA profiling from low-input total RNA. Rna 2007; 13: 151–159. 17105992 10.1261/rna.234507 PMC1705746

