# Supplementary figures and images for: RBM24 suppresses cancer progression by upregulating miR-25 to target MALAT1 in nasopharyngeal carcinoma
Source: Cell Death Dis. 2016 Sep 1;7(9):e2352–. doi: 10.1038/cddis.2016.252 (PMC5059856; doi:10.1038/cddis.2016.252)

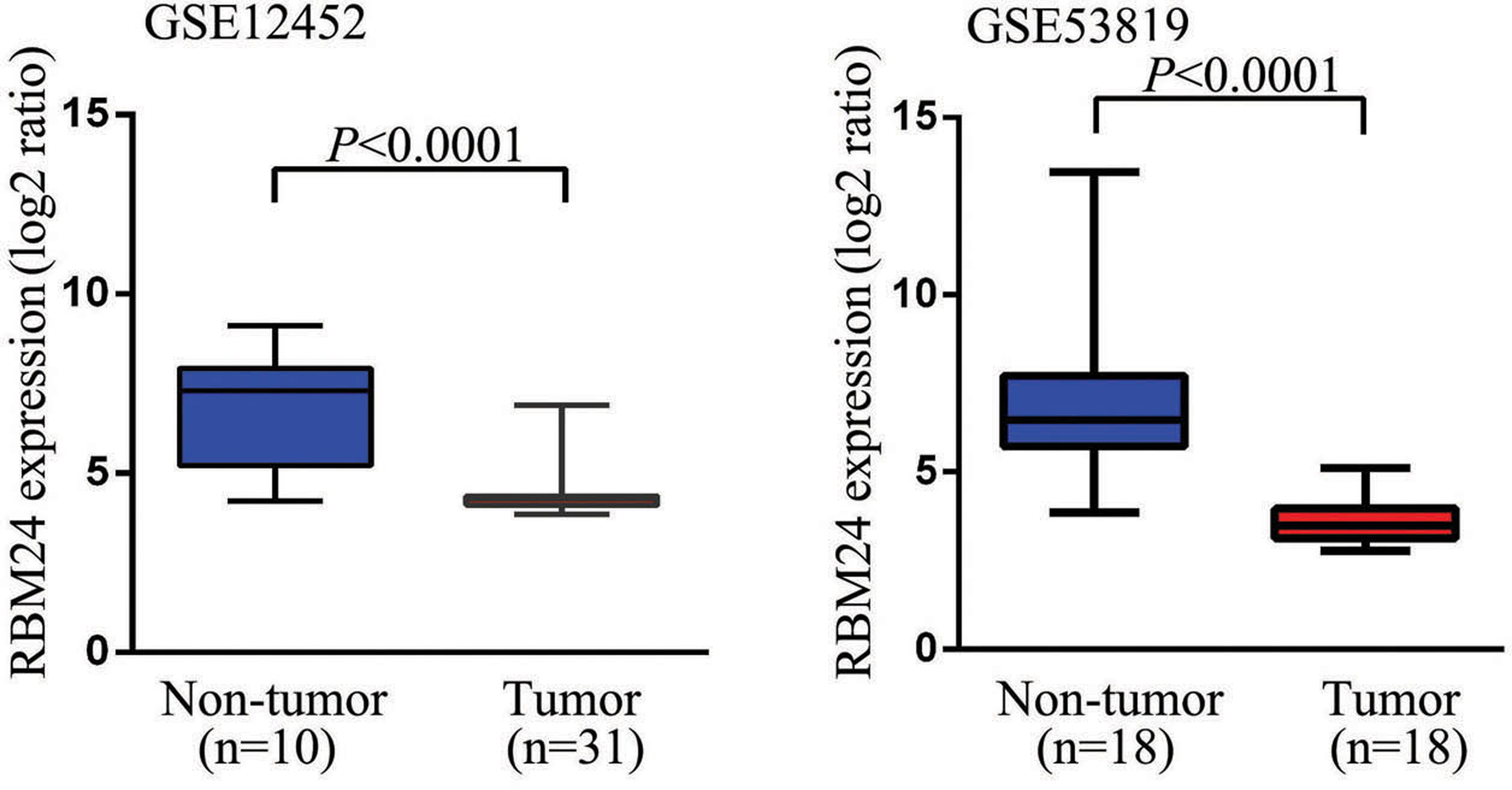

Supplement: Supplementary Figure 1 [file cddis2016252x1.tif]

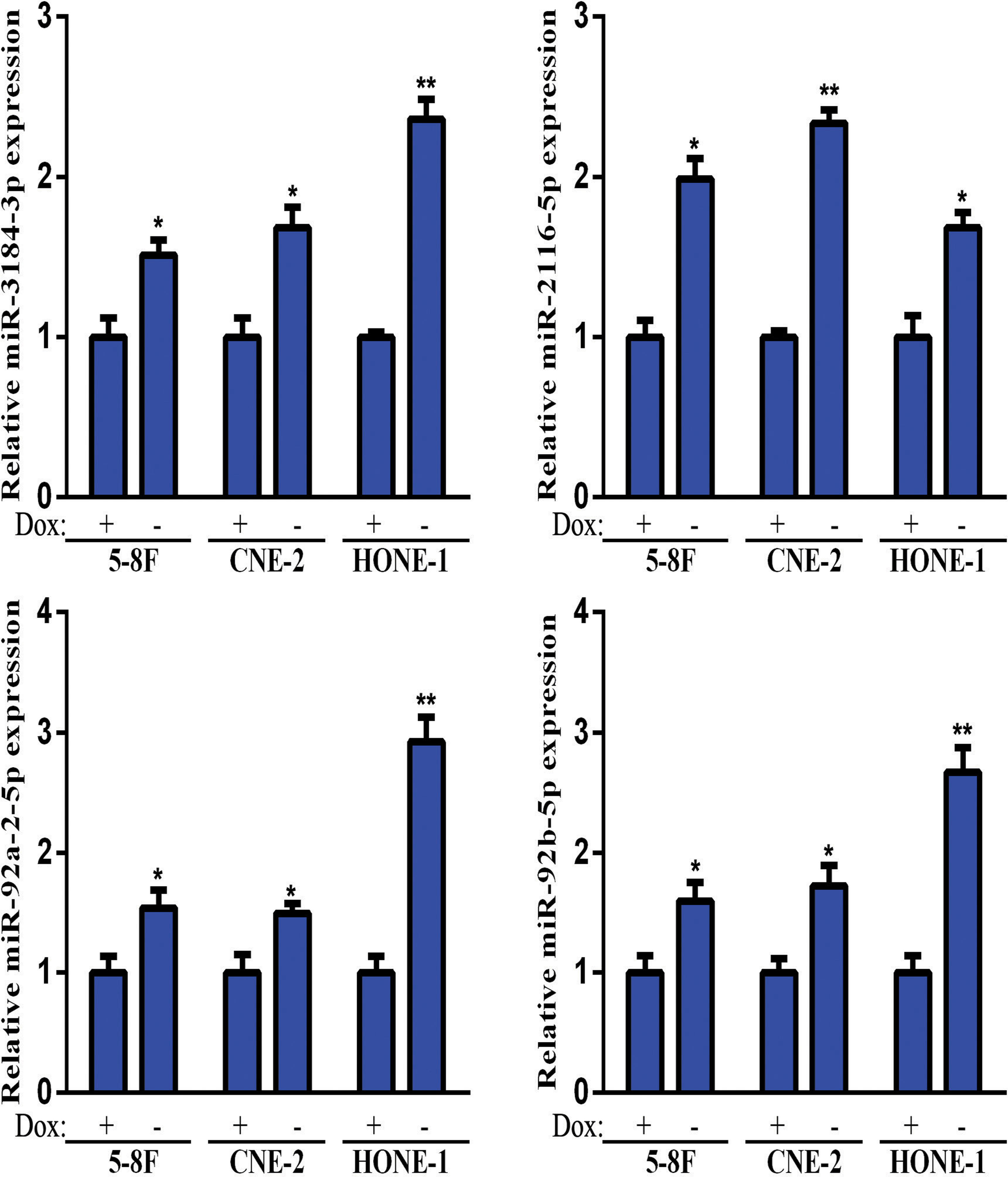

Supplement: Supplementary Figure 2 [file cddis2016252x2.tif]

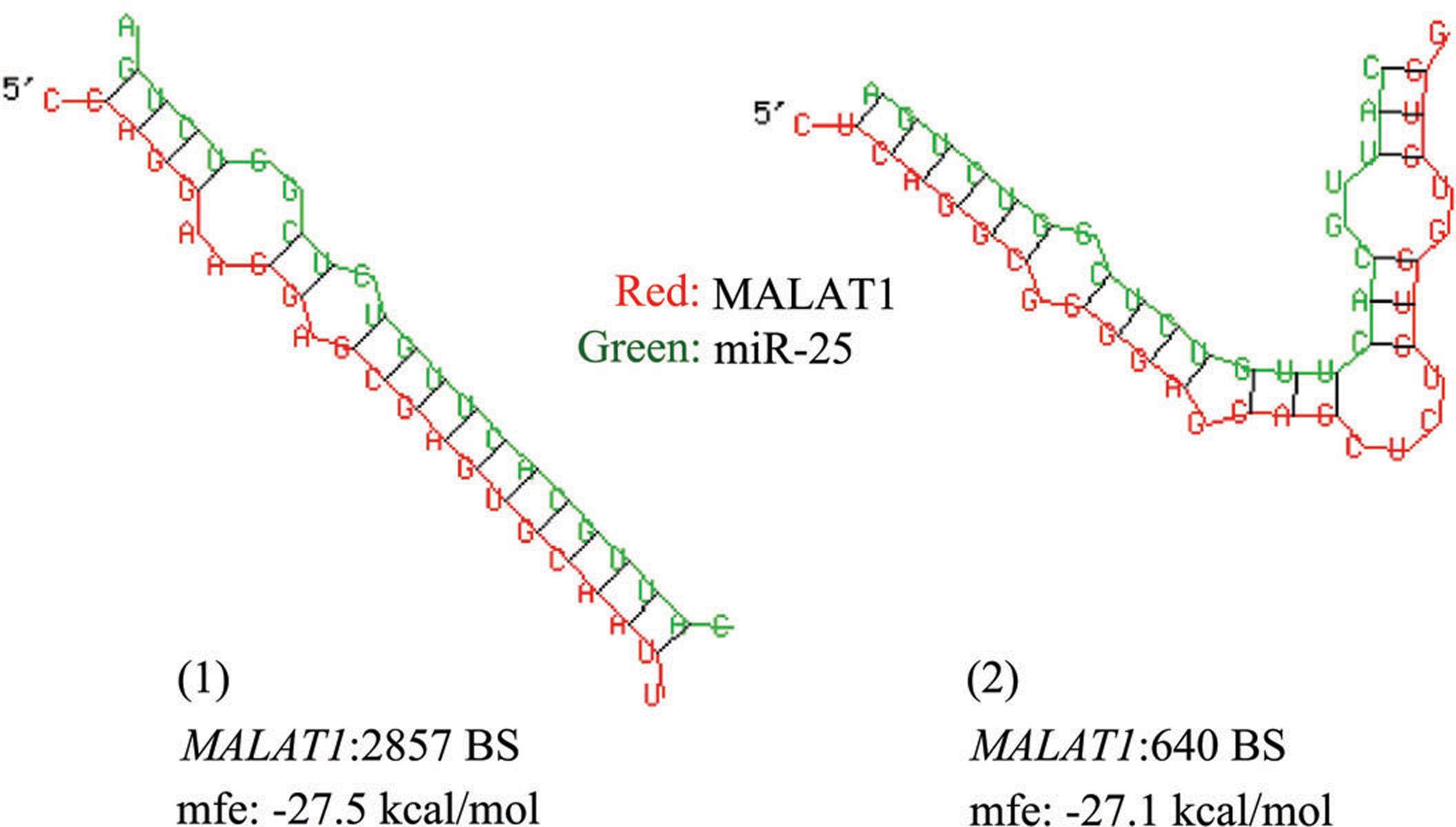

Supplement: Supplementary Figure 3 [file cddis2016252x3.tif]

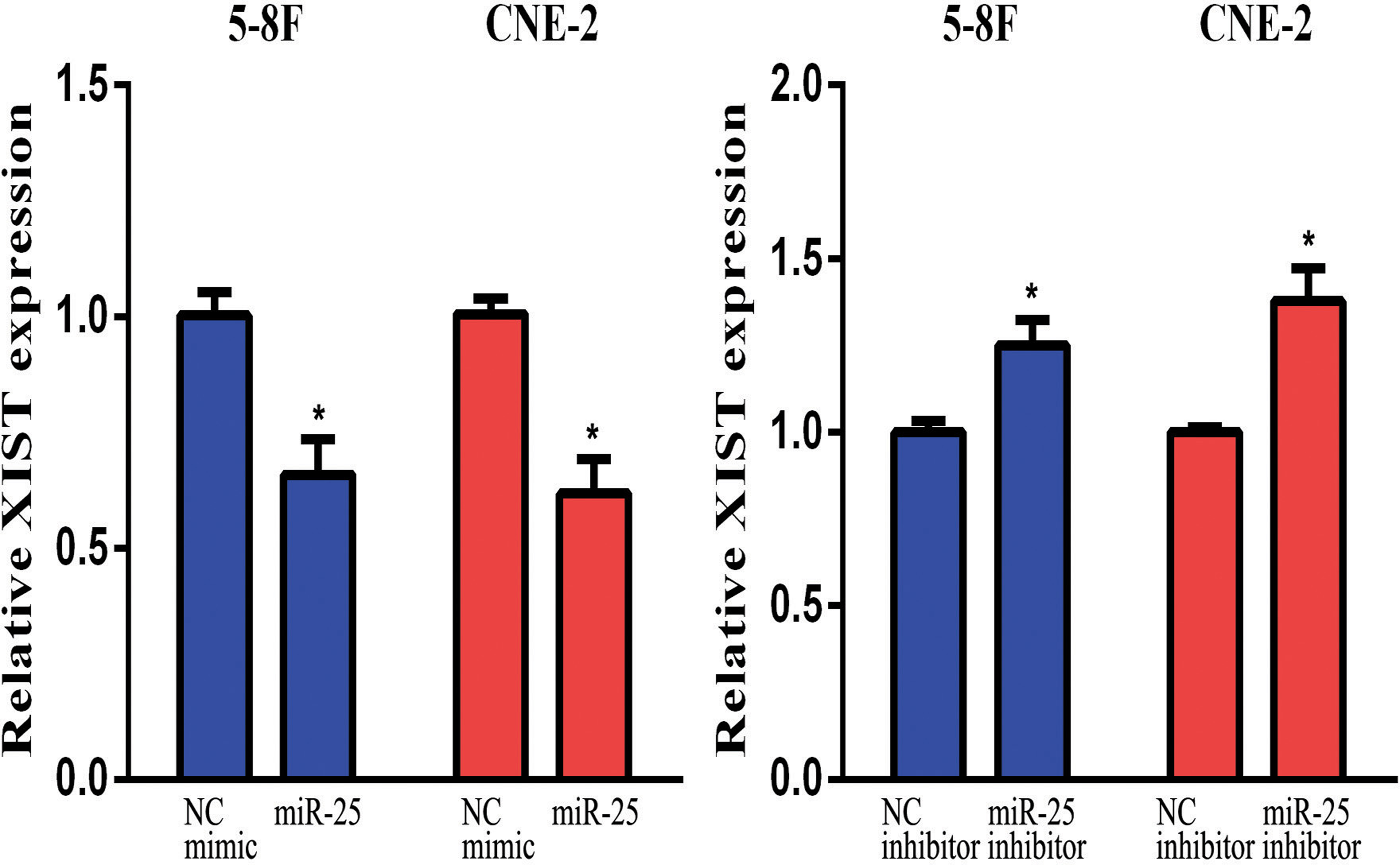

Supplement: Supplementary Figure 4 [file cddis2016252x4.tif]

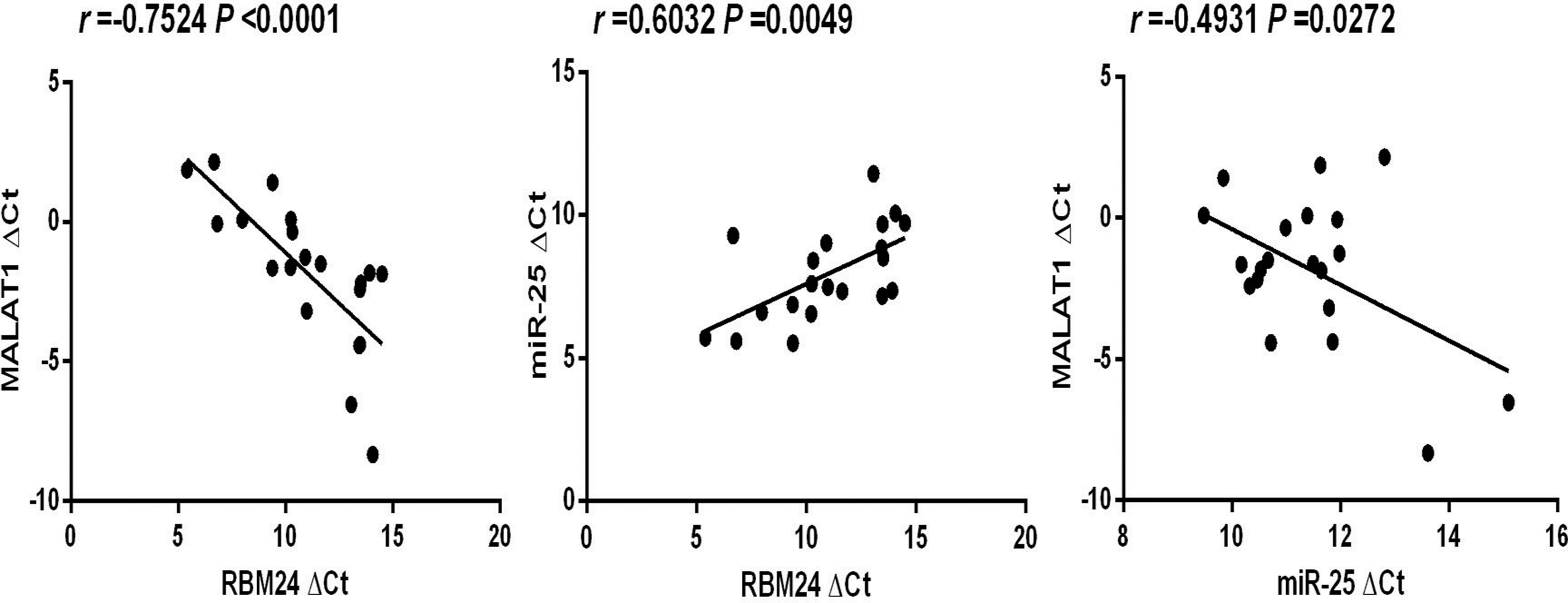

Supplement: Supplementary Figure 5 [file cddis2016252x5.tif]

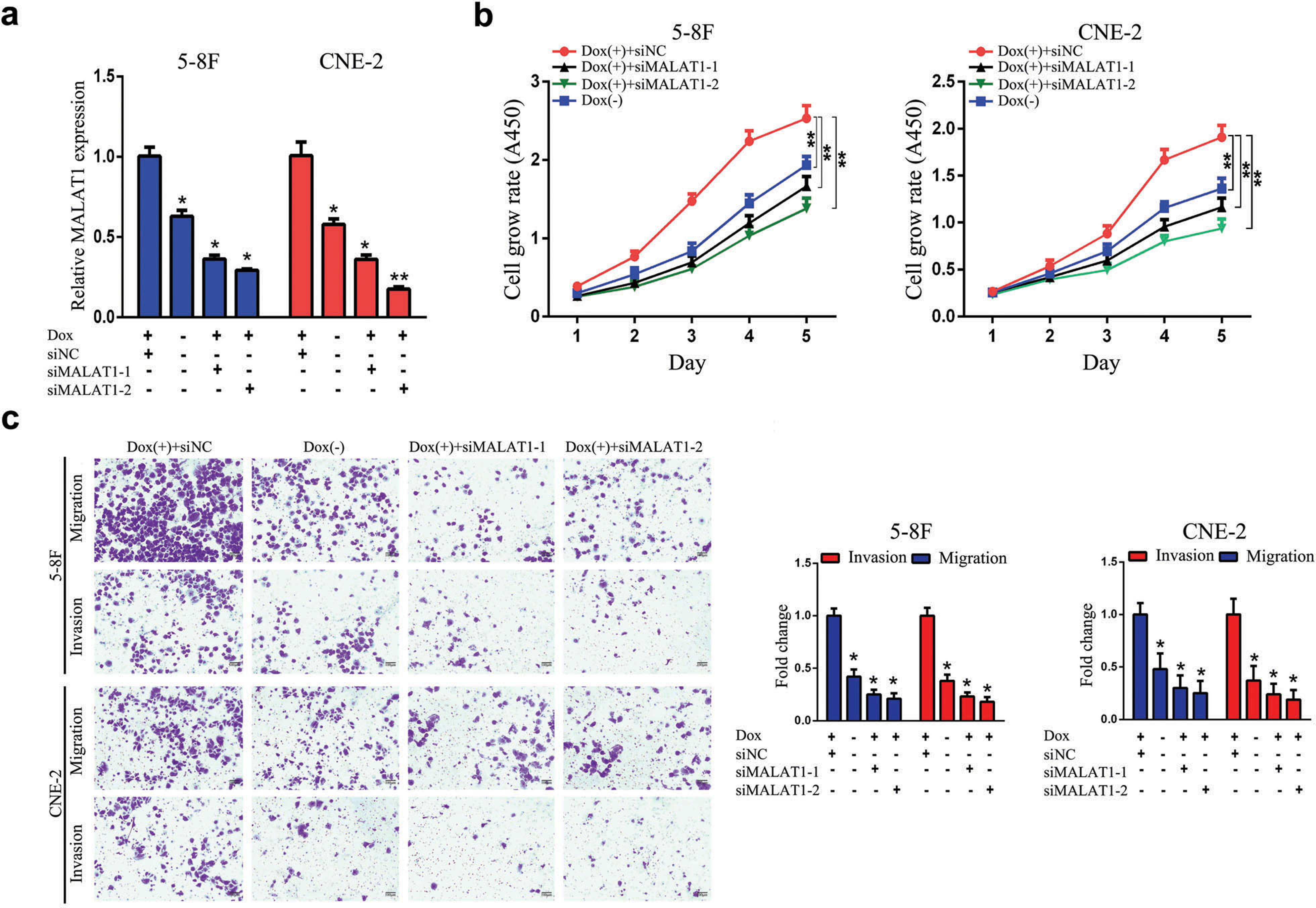

Supplement: Supplementary Figure 6 [file cddis2016252x6.tif]
