# Supplementary material for: RBM24 suppresses cancer progression by upregulating miR-25 to target MALAT1 in nasopharyngeal carcinoma
Source: Cell Death Dis. 2016 Sep 1;7(9):e2352–. doi: 10.1038/cddis.2016.252 (PMC5059856; doi:10.1038/cddis.2016.252)
Supplement: Supplementary Information [file cddis2016252x7.docx]

**Supplementary Figure Legends**

**Supplementary Figure 1. Downregulation of RBM24 in NPC.** The differential expression of RBM24 between NPC tumors and non-cancerous nasopharyngeal tissues was determined from the published expression datasets GSE12452 and GSE53819 of the NCBI GEO database. The middle lines in the colored boxes indicate the medians, the box edges indicate the 25th/75th percentiles, and the whiskers show the 0th/100th percentiles.

**Supplementary Figure 2. Induction of RBM24 expression increases the miRNAs levels in NPC cells.** QRT-PCR analysis of the expression of miR-3184-3p, miR-2116-5p, miR-92a-2-5p and miR-92b-5p in the three Tet-Off-inducible RBM24-stable NPC cell lines treated with or without doxycycline, respectively (*p < 0.05 and **p < 0.01, Student’s *t* test).

**Supplementary Figure 3. The miR-25 target sequences in the MALAT1 sequence.** The predicted miR-25 target sequences in the MALAT1 sequence obtained via RNA-RNA hybrid analysis.

**Supplementary Figure 4. XIST expression is regulated by miR-25 in NPC cells.** QRT-PCR analysis of XIST expression in 5-8F and CNE-2 Tet-Off-inducible RBM24-stable cells that were transfected with miR-25 inhibitor (100 nM) or NC inhibitor after the removal of doxycycline for 24h (*p< 0.05, Student’s *t* test).

**Supplementary Figure 5.Correlation analysis among expression of RBM24, miR-25, and MALAT1 RNA levels in NPC tissues.** Pearson correlation analysis of the RBM24, miR-25, and MALAT1 RNA levels in the NPC tissues, as represented by Pearson R scores (n=20, R < 0 denotes negative correlation).

**Supplementary Figure 6. Knockdown of MALAT1 suppresses NPC cellular growth, migration, and invasion.**(**a**) QRT-PCR analysis of MALAT1 expression in 5-8F and CNE-2 Tet-Off-inducible RBM24-stable cells that were transiently transfected with siMALAT1(50 nM) or siNC after treatment with doxycycline or removal of doxycycline for 24 h (**p< 0.01, Student’s *t* test). (**b**) CCK8 assay ofproliferation of 5-8F and CNE-2 Tet-Off-inducible RBM24-stable cells treated as in (**a**) . (**c**) Transwell assay showing the migration and invasion of 5-8F and CNE-2 Tet-Off-inducible RBM24-stable cells treated as in (**a**). Migrating and invaded cells were fixed and stained with crystal violet (magnification, 100×). The number of migrating andinvadedcells were calculated and are depicted in the bar chart. All data are shown as the mean ± SEM of 3 independent experiments (*p< 0.05 and **p< 0.01, Student’s *t* test).

**Supplementary Materials and Methods**

**Sample information and RNA sequencing**

12 NPC tissues and 3 non-cancerous nasopharyngeal tissues were acquired from Sun Yat-Sen University Cancer Center’s Tumor Tissue Bank. Tissues were obtained from surgery and snap-frozen and then immediately immersed in RNA later reagent (Ambion, TX, USA) overnight at 4°C. These tissues were stored at -80°C prior to RNA extraction. Primary human nasopharyngeal carcinoma epithelial cell lines (SUNE2 and SHEN) were maintained in RPMI 1640 medium (Life Technologies, Carlsbad, CA) supplemented with 10% fetal bovine serum (FBS) in a humidified 5% CO_2_ incubator at 37°C. Normal nasopharyngeal epithelial cell line (NPEC1) was cultured in keratinocyte/serum-free medium (Invitrogen).Total RNA was isolated from each cell linein duplicate usingTRIzol reagent (Invitrogen).For each sample, 0.5μg of total RNA was usedfor library construction.

For RNA sequencing, paired-end libraries including NPEC1, SUNE-2, SHEN, 3 pools of non-cancerous nasopharyngeal epithelial tissues and 12 NPC tissues were prepared according to the protocol provided by Illumina (San Diego, CA, USA) with the mRNA-seq Sample Prep Kit (Illumina).The expression abundance for each gene was measured by RPKM (number of exon reads mapped per kilobase per million mapped).For RNA-seq data analysis, we performed differential gene and transcript expression analysis using TopHat and Cufflinks (Trapnell C,*et al*.*Nat Protoc* 2012.7(3):562-78).

This study was approved by the Institute Research Medical Ethics Committee of Sun Yat-Sen University Cancer Center, and written informed consent was obtained from all patients.
